# Electroacupuncture Ameliorates Cognitive Impairment in Vascular Dementia Rats: Potential Involvement of the Astrocyte–Synapse Axis

**DOI:** 10.1002/brb3.71537

**Published:** 2026-06-08

**Authors:** Sijing Guo, Qian Liu, Shihao Lin, Taotao Zhou, Qin Hu, Zhongsheng Tang

**Affiliations:** ^1^ School of Basic Medicine Guizhou University of Traditional Chinese Medicine Guiyang China; ^2^ Department of Neurosurgery Ren Ji Hospital Shanghai Jiao Tong University School of Medicine Shanghai China; ^3^ Anatomy Teaching and Research Office Guizhou University of Traditional Chinese Medicine Guiyang China

**Keywords:** astrocyte–synapse axis, electroacupuncture, neuroinflammation, synaptic structural integrity, vascular dementia

## Abstract

**Introduction:**

This study investigated whether electroacupuncture at “Zhisanzhen” (EA‐ZSZ) alleviates cognitive impairment in vascular dementia (VD) rats by regulating the astrocyte–synapse axis, reshaping the hippocampal secretory microenvironment, and preserving synaptic structural integrity.

**Methods:**

Sprague‐Dawley rats were divided into sham, VD, VD+EA, and VD+Nim (nimodipine) groups. The VD model was established via a modified bilateral common carotid artery occlusion (2‐VO). After 21 days, learning and memory were evaluated using the Morris water maze. Hippocampal CA1 histopathology and ultrastructure were assessed by H&E and transmission electron microscopy (TEM). RNA‐seq identified differentially expressed genes (DEGs) and pathways. GFAP, BDNF, bFGF, and cytokines (IL‐1β, IL‐6, and TNF‐α) were measured via immunofluorescence, Western blot, and ELISA.

**Results:**

Compared to sham group, VD rats exhibited cognitive impairment, neuronal disorganization, and synaptic disruption. EA‐ZSZ markedly alleviated cognitive deficits, outperforming nimodipine in spatial learning/memory, and restored synaptic ultrastructure (showing clearer clefts and increased vesicle abundance on TEM). RNA‐seq showed that EA‐ZSZ normalized pathways associated with cytokine‐cytokine receptor interactions and synaptic signaling, restoring key “reversal genes” (*Mdk*, *Homer1*, and *Npas4*). These changes were accompanied by reduced GFAP, upregulated hippocampal BDNF/bFGF, and suppressed systemic cytokines (IL‐1β, IL‐6, and TNF‐α).

**Conclusion:**

EA‐ZSZ exerts significant neuroprotective effects in VD rats by modulating the astrocyte–synapse axis, suppressing neuroinflammation, and enhancing neurotrophic/structural support within the hippocampal microenvironment. This highlights its multi‐target advantage over monotherapies in repairing damaged synaptic architecture, supporting its clinical use.

## Graphical abstract

Electroacupuncture significantly ameliorates cognitive impairment and synaptic damage in vascular dementia rats, showing specific advantages over nimodipine. These neuroprotective effects are closely associated with modulating the astrocyte–synapse axis to suppress neuroinflammation and enhance neurotrophic support.

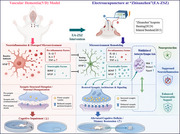

## 1 Introduction

Vascular dementia (VD) is one of the most common forms of dementia and a major contributor to vascular cognitive impairment, imposing a substantial global health burden (Sachdev et al. 2026; Cai et al. 2025; Wong and Chui 2022; O'Brien and Thomas 2015). Despite growing recognition of its clinical importance, currently available therapies remain limited in efficacy, highlighting an urgent need for novel interventions (Wong and Chui 2022; O'Brien and Thomas 2015; VasCog‐2‐WSO Criteria Consortium et al. 2025). Accumulating evidence indicates that cerebrovascular injury, chronic cerebral hypoperfusion, blood‐brain barrier disruption, and persistent neuroinflammatory responses are central pathological features of VD, collectively promoting neuronal loss and synaptic dysfunction (Sachdev et al. 2026; Hosoki et al. 2023; Tian et al. 2022). Because synaptic integrity is essential for neuronal information processing and memory formation, synaptic injury is increasingly recognized as a key substrate of cognitive impairment in VD (Tian et al. 2022; Kalaria 2018).

The hippocampus, particularly the CA1 subfield, is highly vulnerable to ischemic and hypoperfusion‐related injury and plays a pivotal role in learning and memory. In VD, structural and functional abnormalities in hippocampal CA1, including dendritic spine loss, impaired synaptic plasticity, and reduced long‐term potentiation, are closely associated with cognitive decline (Wu et al. 2025). In parallel, astrocytes (ASs) are critical regulators of the synaptic microenvironment, supporting neuronal metabolism, maintaining neurotransmitter homeostasis, and modulating synaptic transmission under physiological conditions (Barros et al. 2024). However, under pathological conditions, reactive ASs may adopt maladaptive phenotypes that exacerbate inflammatory signaling, disrupt the AS–synapse axis, and contribute to synaptic degeneration (Escartin et al. 2021). These findings suggest that AS dysfunction may be an important mechanism underlying synaptic failure in VD.

Acupuncture has attracted increasing attention as a potential intervention for VD, and existing clinical evidence suggests that it may improve cognitive performance, particularly learning and memory (Su et al. 2021; Wen et al. 2022). Among different acupuncture protocols, Zhisanzhen, consisting of Shenting (DU24) and bilateral Benshen (GB13), has been used in cognitive and neuropsychiatric disorders because of its simplicity, low discomfort, and clinical accessibility (J. Zhang et al. 2019; N. Zhang et al. 2025; Jianyi et al. 2023). Electroacupuncture (EA) offers standardized and quantifiable stimulation parameters and may facilitate reproducibility and mechanistic investigation compared with manual acupuncture (Ulloa 2021; Zheng et al. 2020). Preclinical and mechanistic studies have shown that EA can modulate inflammatory signaling and exert neuroprotective effects (Zheng et al. 2020; Liu et al. 2021; Napadow et al. 2005). However, whether electroacupuncture at Zhisanzhen (EA‐ZSZ) improves cognitive dysfunction in VD through regulation of AS activation and synaptic integrity remains unclear. Therefore, in this study, we established a VD rat model using modified permanent bilateral common carotid artery occlusion (2‐VO) and combined RNA sequencing (RNA‐seq) with ultrastructural and molecular analyses to investigate whether EA‐ZSZ alleviates cognitive deficits by suppressing AS overactivation and neuroinflammation while preserving synaptic structure in the hippocampal CA1 region.

## 2 Materials and Methods

### 2.1 Animals

Specific pathogen‐free (SPF) healthy male Sprague‐Dawley (SD) rats (aged: 6–8 weeks; weighing: 250 ± 30 g) were purchased from Changsha Tianqin Experimental Animal Breeding Co. Ltd. (Production License No. SCXK (Xiang) 2022‐0011). The rats were housed in the Dongyuan Laboratory Animal Facility of Guizhou University of Traditional Chinese Medicine under controlled conditions: a temperature 25 ± 1*°*C, a relative humidity 55% ± 5%, and a 12 h/12 h light‐dark cycle, with free access to food and water. After a 1‐week acclimatization period, the rats were screened using the Morris water maze (MWM) test, and those exhibiting immobile or floating behaviors were excluded. Subsequently, 12 rats were randomly assigned to the sham group using a random number table, while the remaining rats were used to establish the VD model. Thirty‐six successfully modeled rats were then randomly divided (via a random number table) into three groups (*n* = 12 per group): the VD group, the nimodipine (VD+Nim) group, and the electroacupuncture (VD+EA) group. This study was approved by the Animal Ethics Committee of Guizhou University of Traditional Chinese Medicine (Ethics Approval No. 2024077).

### 2.2 Induction of VD

The VD model was established using a modified permanent 2‐VO method (Fu et al. 2024). Prior to surgery, rats were fasted for 12 h and anesthetized via intraperitoneal injection of 60 mg/kg sodium pentobarbital (Flecknell 2016). A ventral midline incision was made in the neck. The common carotid arteries were exposed and separated from the vagus nerve. Carotids were occluded with a 1‐week interval between interventions; the right common carotid was the first to be processed, and the left one was occluded 1 week later. Seven days post‐surgery, the model was validated using the MWM test. The baseline value was defined as the baseline value of the sham group on Day 4. To ensure the reliability of the VD model and minimize individual variability, animals exhibiting a change in mean escape latency of less than 20% compared to their baseline values were excluded from the study.

### 2.3 EA‐ZSZ Intervention and Nimodipine Treatment

The acupuncture treatments were performed by an experienced acupuncturist between 9:00 a.m. and 11:00 a.m. EA stimulation was applied at the “Zhisanzhen” acupoints, including Shenting (GV24) and bilateral Benshen (GB13). Single‐use disposable needles (0.25 mm × 13 mm, Suzhou Huatuo Brand) were used for treatment. In the VD+EA group, the needles were inserted to a depth of 1–2 mm at the three acupoints. After insertion, two acupoints were selected for EA stimulation (SDZ‐II, Huatuo brand, China) to minimize acupoint tolerance. Electrical stimulation (disperse‐dense wave, 2/15 Hz frequency, 1 mA intensity) was performed for 20 min/day for 21 days (Guo et al. 2018; She et al. 2015; Chen et al. 2025). In the VD+Nim group, rats received nimodipine (20 mg/kg/day, once daily) for 21 days (Yang et al. 2024; López‐Arrieta and Birks 2002).

### 2.4 Morris Water Maze Test

Following the treatment period, the MWM test (Guangzhou Yongnuo, China) was conducted to evaluate the spatial learning and memory of rats, as previously described (Zheng et al. 2020). The experimental procedures were divided into three phases: visible platform trials, hidden platform trials, and spatial probe trials. The water temperature was strictly maintained at 23 ± 2°C. On Day 15 of treatment, a visible platform trial was performed to exclude any interference from sensory or motor deficits. The platform was placed 1 cm above the water surface in the first quadrant and marked with a conspicuous flag. Rats were trained three times to locate the platform. Starting from Day 16 to Day 20 of treatment, the hidden platform (spatial acquisition) test was conducted for five consecutive days. The platform was submerged 1 cm below the water surface. Rats underwent three trials per day, each starting from a different quadrant facing the wall. The maximum duration for each trial was 120 s. The time taken to reach the platform was recorded. If a rat failed to locate the platform within 120 s, it was guided to the platform and allowed to stay for 15 s, and the latency was recorded as 120 s. The average escape latency across these 5 days was calculated to represent spatial learning ability. On Day 21 of treatment, the spatial probe trial was conducted to evaluate memory retention. The platform was removed from the pool. Rats were released into the water from the third quadrant. The number of times the rats crossed the original platform location within 60 s was recorded and analyzed.

### 2.5 RNA Sequencing and Analysis

#### 2.5.1 RNA Extraction, Library Construction, and Sequencing

Total RNA was extracted from hippocampal tissue using TRIzol reagent (Thermo Fisher Scientific, cat. 15596018) according to the manufacturer's instructions. RNA concentration and purity were assessed using an Agilent 2100 Bioanalyzer and the RNA 6000 Nano LabChip Kit (Agilent Technologies, CA, USA; cat. 5067‐1511). Only RNA samples with an RNA integrity number (RIN) > 7.0 were used for library preparation.

For each sample, mRNA was isolated from 5 µg of total RNA using Dynabeads Oligo (dT) (Thermo Fisher Scientific, CA, USA) with two rounds of purification. The purified mRNA was fragmented into short fragments under elevated temperature in the presence of divalent cations using the Magnesium RNA Fragmentation Module (NEB, cat. E6150, USA) at 94°C for 5–7 min. The fragmented RNA was reverse‐transcribed into first‐strand cDNA using SuperScript II Reverse Transcriptase (Invitrogen, cat. 1896649, USA). Second‐strand cDNA was synthesized using *Escherichia coli* DNA polymerase I (NEB, cat. M0209, USA), RNase H (NEB, cat. M0297, USA), and dUTP Solution (Thermo Fisher Scientific, cat. R0133, USA).

The resulting cDNA fragments were end‐repaired, A‐tailed, and ligated to dual‐index adapters with T overhangs. After size selection using AMPure XP beads, the dUTP‐marked second‐strand cDNA was treated with heat‐labile uracil‐DNA glycosylase (UDG; NEB, cat. M0280, USA). The adapter‐ligated products were then amplified by PCR under the following conditions: initial denaturation at 95°C for 3 min; 8 cycles of denaturation at 98°C for 15 s, annealing at 60°C for 15 s, and extension at 72°C for 30 s; and a final extension at 72°C for 5 min. The average insert size of the final cDNA libraries was 300 ± 50 bp. Paired‐end sequencing (2 × 150 bp) was performed on an Illumina NovaSeq 6000 platform according to the manufacturer's protocol.

#### 2.5.2 Sequencing Data Processing and Quality Control

The cDNA libraries prepared from pooled RNA derived from rat (*Rattus norvegicus*) hippocampal tissue were sequenced on the Illumina NovaSeq 6000 platform using a paired‐end RNA‐seq strategy. Raw reads were filtered using Cutadapt (https://cutadapt.readthedocs.io/en/stable/; version 1.9) to remove adapter sequences, polyA/polyG reads, reads containing more than 5% ambiguous nucleotides (N), and reads with more than 20% low‐quality bases (*Q*‐score ≤ 20). Clean reads were then evaluated using FastQC (http://www.bioinformatics.babraham.ac.uk/projects/fastqc/; version 0.11.9) for quality metrics including Q20, Q30, and GC content.

#### 2.5.3 Alignment to the Reference Genome

Clean reads were aligned to the rat (*R. norvegicus*) reference genome using HISAT2 (https://daehwankimlab.github.io/hisat2/; version 2.2.1) with default parameters. HISAT2 is a splice‐aware aligner for RNA‐seq data that maps reads to the reference genome and identifies exon–exon splice junctions by constructing a database of candidate splice sites and validating them using unmapped reads.

#### 2.5.4 Quantification of Gene Expression

Mapped reads from each sample were assembled into transcripts using StringTie (http://ccb.jhu.edu/software/stringtie/; version 2.1.6) with default parameters. Transcript assemblies from all samples were merged using gffcompare (http://ccb.jhu.edu/software/stringtie/gffcompare.shtml; version 0.9.8) to generate a comprehensive transcriptome annotation. Transcript abundance was estimated using StringTie and Ballgown (http://www.bioconductor.org/packages/release/bioc/html/ballgown.html), and gene expression levels were quantified as fragments per kilobase of transcript per million mapped reads (FPKM).

#### 2.5.5 Differential Expression Analysis

Differential expression analysis between the two groups was performed using DESeq2. Genes with a *p*‐value < 0.05 and an absolute fold change ≥ 2 were considered differentially expressed genes (DEGs). The DEGs were then subjected to Gene Ontology (GO) and Kyoto Encyclopedia of Genes and Genomes (KEGG) enrichment analyses.

#### 2.5.6 GO Enrichment Analysis

GO enrichment analysis was performed to identify GO terms significantly enriched among the DEGs relative to the genomic background. DEGs were mapped to GO terms in the Gene Ontology database (http://www.geneontology.org/), and the number of genes associated with each term was calculated. Significantly enriched GO terms were identified using a hypergeometric test.

#### 2.5.7 KEGG Pathway Enrichment Analysis

KEGG pathway enrichment analysis was conducted to identify significantly enriched metabolic and signal transduction pathways among the DEGs compared with the whole‐genome background. This analysis was performed to further explore the biological functions and potential pathways associated with the DEGs.

### 2.6 Hematoxylin‐Eosin Staining and Immunofluorescence Staining

Hippocampal tissue sections were baked at 70°C for 30 min, dewaxed in xylene (twice, 5 min each), and rehydrated through a graded ethanol series (100%, 90%, 80%, and 70%, 5 min each), followed by distilled water rinsing (5 min). Sections were stained with hematoxylin for 5 min, rinsed, differentiated in 1% hydrochloric acid alcohol for 1–3 s, and blued in running water for 5 min. Eosin (0.5%) was applied for 15 s, followed by distilled water rinsing. Dehydration was performed in ascending ethanol concentrations (80%, 90%, 95%, and 100%), cleared in xylene (twice, 5 min each), and mounted with neutral resin. Morphological changes were observed under a microscope, and images were captured for analysis.

Brain sections were dewaxed, antigen‐retrieved in citrate buffer (20 min), permeabilized with 0.5% Triton X‐100 (30 min), and blocked with 3% hydrogen peroxide (25 min). After PBS washes, sections were blocked with 5% BSA (30 min), incubated with primary antibody (GFAP, CST3670S, USA) at 4°C overnight, washed, and incubated with fluorescent secondary antibody (60 min, room temperature). Images were captured using a confocal microscope (FV3000, Olympus, Japan), and fluorescence intensity was analyzed with ImageJ.

### 2.7 Transmission Electron Microscope Imaging

For transmission electron microscope (TEM) assessment, tissue blocks (approximately 1 × 1 × 1 mm^3^) were precisely microdissected from the hippocampal CA1 region. The specimens were fixed in 3% glutaraldehyde for 48 h and post‐fixed in 1% osmium tetroxide for 24 h. Dehydration was conducted using a graded acetone series (30%, 50%, 70%, 80%, 90%, 95%, and three changes of 100%). Tissues were then infiltrated with varying ratios of acetone and Epon‐812 resin (3:1, 1:1, and 1:3) and embedded in pure Epon‐812 resin. Semi‐thin sections were first examined under a light microscope to accurately identify the stratum radiatum of the CA1 region. Subsequently, ultrathin sections (60 nm) were cut, mounted on copper grids, and double‐stained with uranyl acetate (15 min) and lead citrate (2 min).

Synaptic ultrastructure was observed using TEM (JEM‐1400FLASH, JEOL, Japan). To ensure an unbiased evaluation, a systematic random sampling strategy was employed, with three non‐overlapping fields of view randomly captured per section at a standardized magnification. For the quantitative analysis of synaptic morphology, only intact asymmetric synapses were selected. The specific inclusion criteria for a valid synapse required the simultaneous presence of a presynaptic terminal with distinct synaptic vesicles, a clearly identifiable synaptic cleft, and a well‐defined postsynaptic density (PSD).

### 2.8 Western Blot

Hippocampal tissues were homogenized in lysis buffer and centrifuged at 12,000 rpm for 20 min at 4°C. The supernatants were collected, and protein concentrations were measured using a bicinchoninic acid assay kit (Thermo Fisher Scientific, USA). Equal amounts of protein (25 µg per lane) were denatured and separated by 10% SDS‐PAGE using a PAGE Gel Fast Preparation Kit (Yamei, China), followed by transfer onto 0.45 µm PVDF membranes. The membranes were blocked with 5% skim milk for 60 min at room temperature on a shaker at 20 rpm and then incubated overnight at 4°C with primary antibodies against BDNF (ab203573, Abcam, UK; 1:1000), bFGF (IPB0287, Biotechnology, China; 1:1000), GFAP (3670S, Cell Signaling Technology, USA; 1:1000), and β‐actin (AC026, Abclonal, China; 1:1000). After washing with TBST three times for 5 min each, the membranes were incubated with HRP‐conjugated secondary antibody (1:2000) for 1 h at room temperature on a shaker at 20 rpm, followed by three additional washes with TBST. Protein bands were visualized using enhanced chemiluminescence (Yamei, China) and captured with a ChemiDoc XRS+ imaging system (Bio‐Rad, USA). Band intensities were analyzed using ImageJ software, and target protein expression levels were normalized to β‐actin.

### 2.9 ELISA for Serum IL‐1β, IL‐6, and TNF‐α Levels

ELISA was performed according to the manufacturer's instructions for the commercial kits (96 Tests, ZC‐36391; 96 Tests, ZC‐36404; 96 Tests, ZC‐37624; Shanghai Zhuocai Biotechnology Co. Ltd., China). Briefly, all reagents were equilibrated to room temperature for 30 min, and the required microplate strips were removed from the foil pouch. Standard wells and sample wells were prepared separately. Standard solutions of different concentrations (50 µL) were added to the standard wells, while 50 µL of the test sample was added to each sample well; the blank wells received no sample. Except for the blank wells, 100 µL of horseradish peroxidase‐conjugated detection antibody was added to each well. The plate was sealed and incubated at 37°C for 60 min in a water bath or constant‐temperature incubator. After incubation, the liquid was discarded, and the plate was blotted dry on absorbent paper. Each well was filled with washing solution and allowed to stand for 1 min, after which the washing solution was discarded and the plate was blotted dry again. This washing procedure was repeated five times. Subsequently, 50 µL of substrate A and 50 µL of substrate B were added to each well, followed by incubation at 37°C for 15 min in the dark. Then, 50 µL of stop solution was added to each well. Within 10 min, the optical density (OD) of each well was measured at 450 nm using a microplate reader (SpectraMax Plus384, Molecular Devices, China).

### 2.10 Statistical Analysis

Data were analyzed using SPSS 26.0 and plotted with GraphPad Prism 8. Normally distributed data were expressed as mean ± SD. Intergroup comparisons were performed using one‐way ANOVA with LSD‐*t* (equal variance) or Dunnett‐*t* (unequal variance) tests. Intragroup comparisons used repeated‐measures ANOVA. Statistical significance was set at *p *< 0.05.

## 3 Results

### 3.1 EA‐ZSZ Attenuated Spatial Learning and Memory Deficits in VD Rats

**FIGURE 1:**
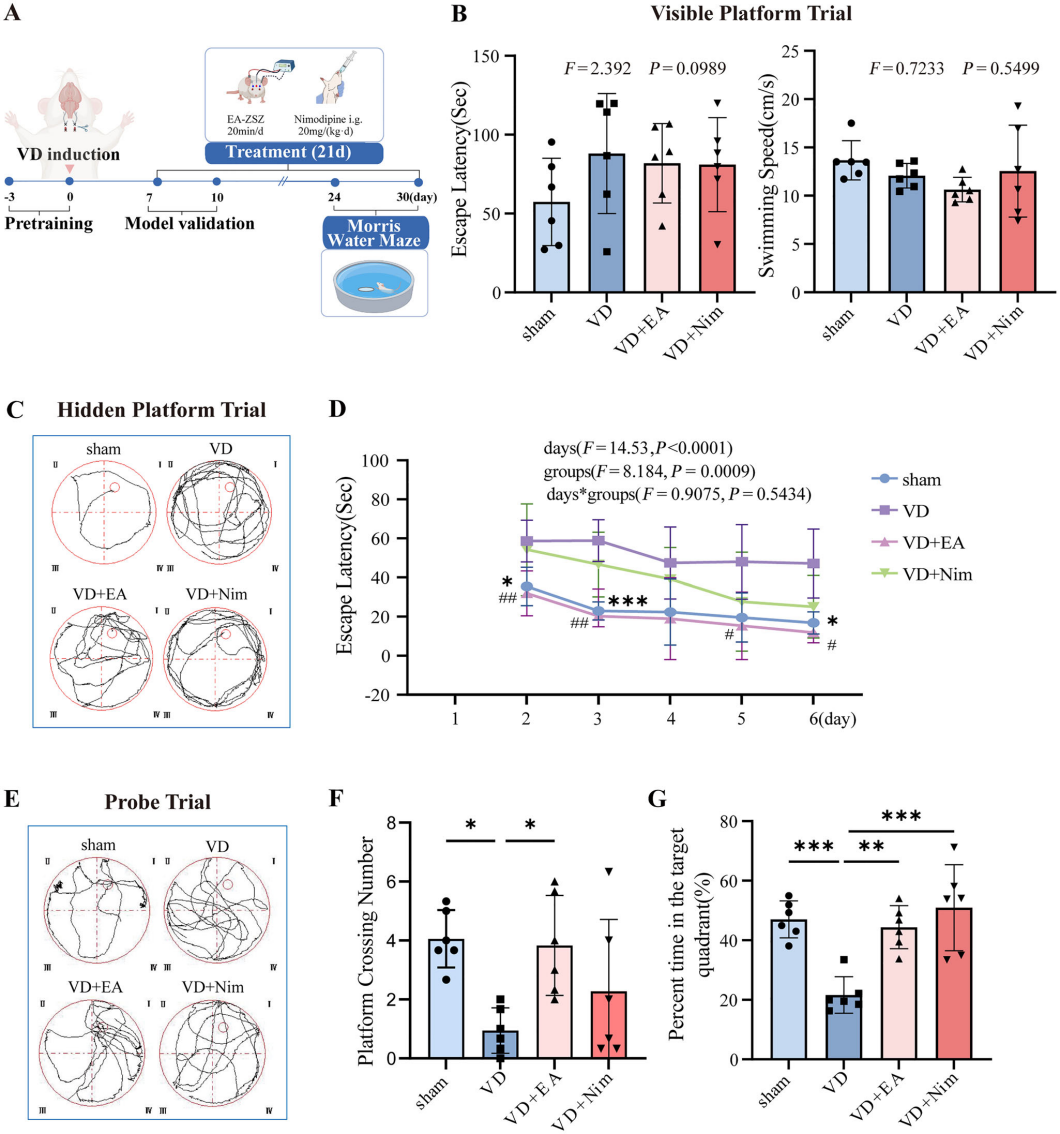
EA‐ZSZ improves learning and memory abilities in VD rats. (A) Schematic diagram of part of the experimental workflow; (B) escape latency and swimming speed during the visible platform test; (C) hidden platform phase trajectory plot; (D) learning curves showing the escape latency to find the hidden platform over five training days (^*^ *p* < 0.05, ^**^ *p* < 0.01, ^***^ *p* < 0.001, compared with the sham group. ^#^ *p* < 0.05, ^##^ *p* < 0.01, compared with the VD group); (E) probe trial phase trajectory; (F) the number of platform crossings from the probe trial on Day 7; (G) the percentage of time spent in the target quadrant from the probe trial on Day 7. Data are expressed as mean ± SD. ^*^ *p* < 0.05, ^**^ *p < *0.01, ^***^ *p* < 0.001, compared with each group; *n* = 6 per group.

We tested whether EA‐ZSZ could improve the spatial learning and memory functions of VD rats via the MWM test (Figure 1A). To exclude potential sensorimotor or visual impairments, a visible platform test was conducted. No significant differences were observed in escape latency or swimming speed among the four groups (Figure 1B), indicating that motor and visual functions were preserved. In the hidden platform trials assessing spatial learning, the VD group exhibited significantly longer escape latencies than the Sham group on Days 2, 3, 5, and 6. Conversely, the VD+EA group showed significantly reduced escape latencies compared to the VD group on these specific days. The escape latency of the VD+Nim group did not differ significantly from the VD groups (Figure 1C,D). In the probe trial, the VD group demonstrated a significant reduction in both the number of platform crossings and the percentage of time spent in the target quadrant compared to the Sham group. Treatment with EA‐ZSZ significantly reversed these deficits, increasing both the crossing number and the time percentage in the target quadrant compared to the VD group. While the VD+Nim group exhibited a significantly higher percentage of time spent in the target quadrant, the number of platform crossings did not differ significantly from the VD group (Figure 1E–G).

### 3.2 EA‐ZSZ Alleviates Histopathological Damage and Promotes Synaptic Ultrastructural Remodeling in the Hippocampal CA1 Region

**FIGURE 2:**
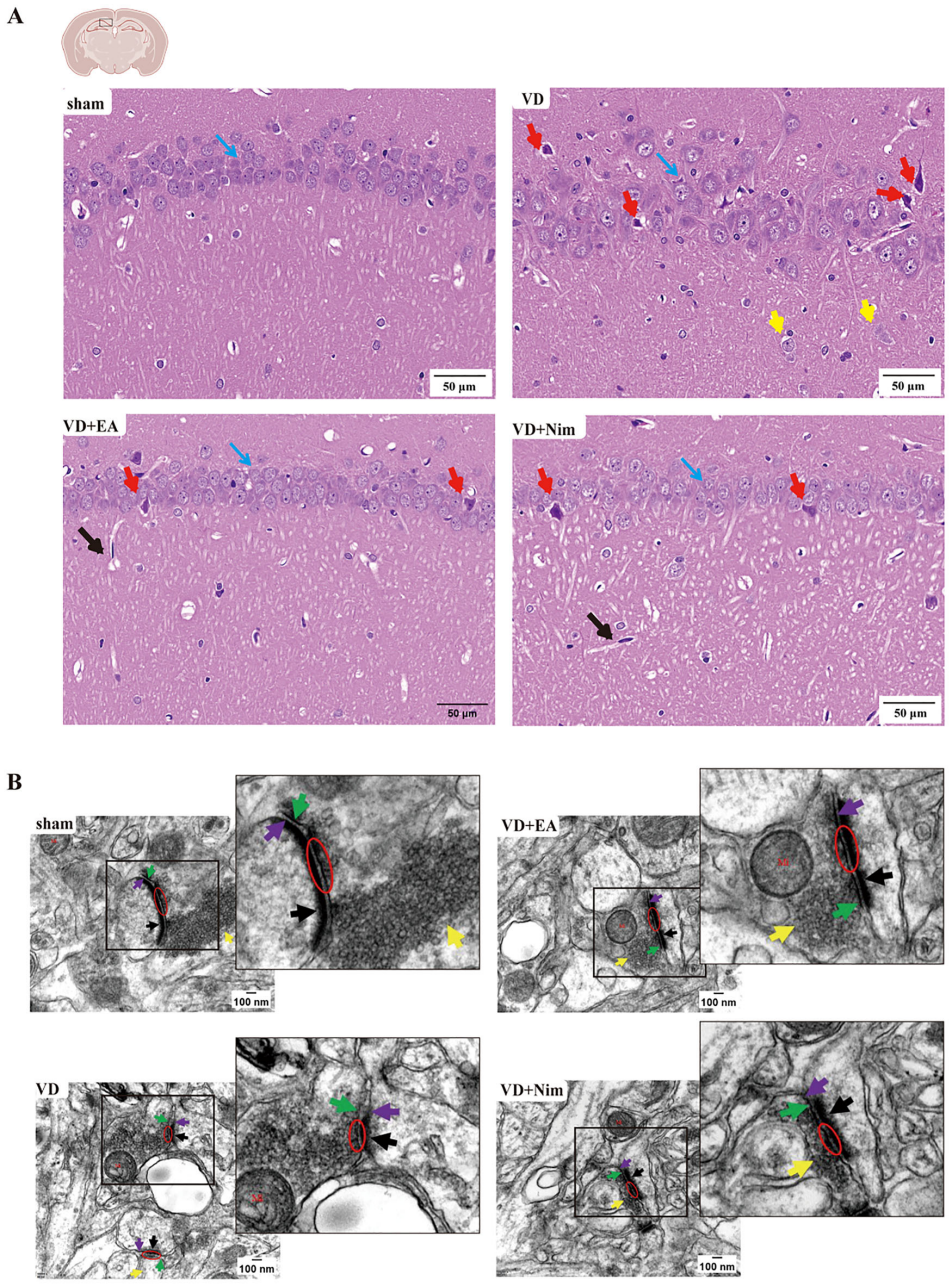
Histological and ultrastructural analysis of the hippocampal CA1 region. (A) Representative H&E staining images of the CA1 region. Neurons (blue arrows), activated astrocytes (red arrows), neutrophils (yellow arrows), and microvessels (black arrows) are indicated. Scale bar = 50 µm. (B) Transmission electron micrographs of neuronal synapses. Structures are labeled as follows: Mi, mitochondria; green arrows, presynaptic membrane; red ellipses, synaptic cleft; purple arrows, postsynaptic membrane; black arrows, postsynaptic density; yellow arrows, synaptic vesicles. Scale bar = 100 nm. *n* = 3 per group.

We evaluated the neuroprotective effects of EA‐ZSZ on histopathology and synaptic ultrastructure. H&E staining demonstrated that VD rats exhibited disordered neuronal arrangement, prominent glial swelling, and inflammatory infiltration compared to the regular, compact neuronal morphology of the Sham group. While both EA‐ZSZ and nimodipine treatments alleviated glial swelling and inflammation, promoting microvessel proliferation, only the EA‐ZSZ group showed a restoration of orderly neuronal arrangement similar to the Sham group (Figure 2A). Furthermore, TEM analysis revealed marked synaptic ultrastructural alterations and impairments in the VD group. These were primarily characterized by reduced presynaptic and postsynaptic contact areas, decreased postsynaptic densities, and synaptic vesicle depletion, accompanied by abnormal changes in synaptic cleft width. Notably, EA‐ZSZ treatment effectively preserved these synaptic structures and the local microenvironment, whereas nimodipine failed to completely reverse these ultrastructural pathologies (Figure 2B). Collectively, these findings suggest that EA‐ZSZ mitigates neuronal and glial death while promoting synaptic structural remodeling in the CA1 region of VD rats.

### 3.3 Transcriptomic Profiling Reveals Coordinated Modulation of Neuroinflammatory and Synaptic Gene Networks by EA‐ZSZ

**FIGURE 3:**
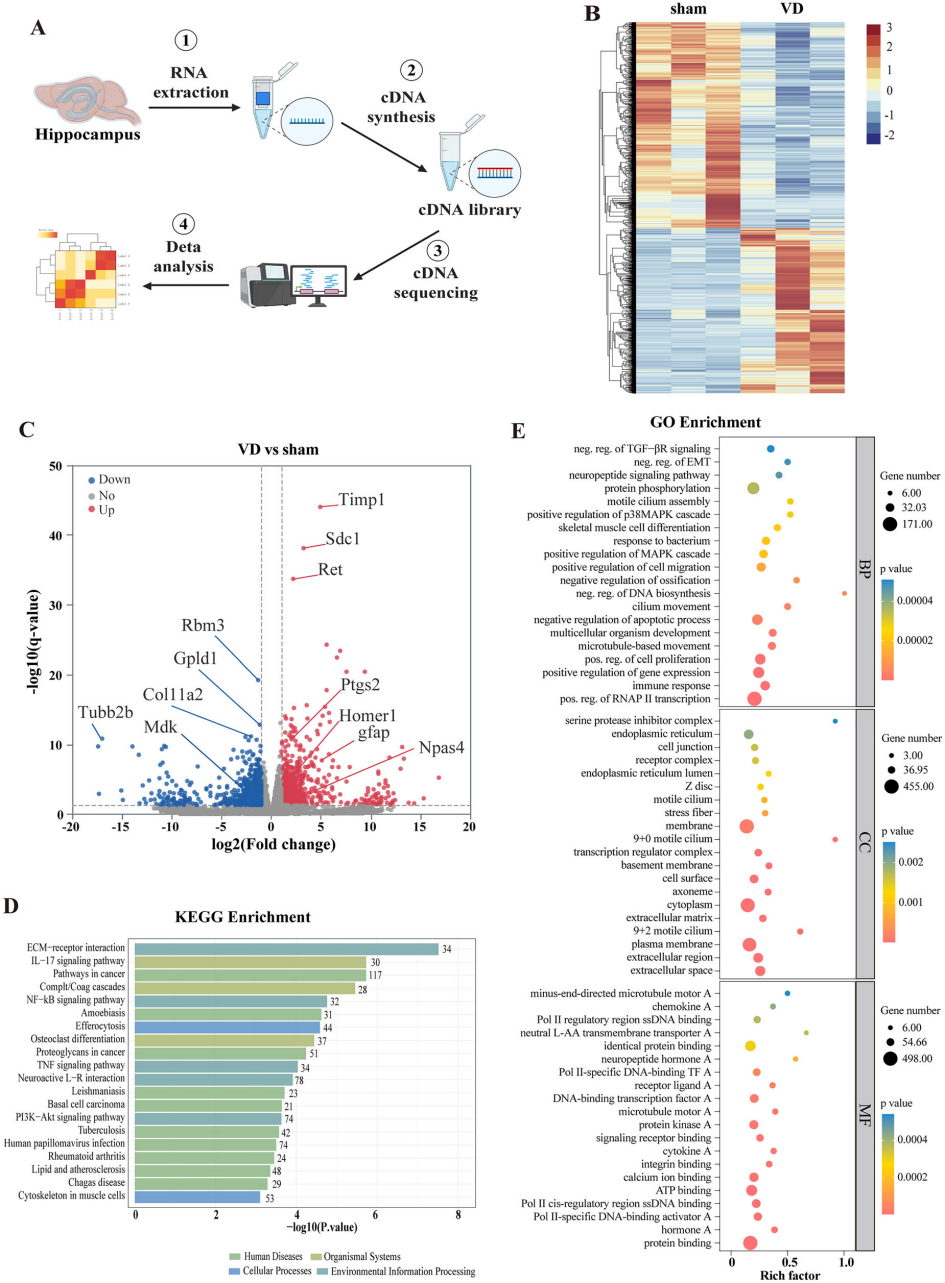
RNA‐seq analysis of transcriptomic alterations in the VD rat hippocampus. (A) Schematic workflow of the RNA‐seq experimental procedure. (B) Heatmap of differentially expressed genes (DEGs) between the Sham and VD groups, ∣log_2_FC∣ ≥ 1 and *p *< 0.05. (C) Volcano plot showing DEGs in the hippocampus between the Sham and VD groups. Significantly downregulated genes are indicated in blue and defined as∣log_2_FC∣ ≤ −1 and *p* < 0.05; significantly upregulated genes are indicated in red and defined as∣log_2_FC∣ ≥ 1 and *p *< 0.05. (D) KEGG pathway enrichment analysis of DEGs between the Sham and VD groups, *p *< 0.05. (E) Gene Ontology (GO) enrichment analysis of DEGs between the Sham and VD groups, *p *< 0.05.

**FIGURE 4:**
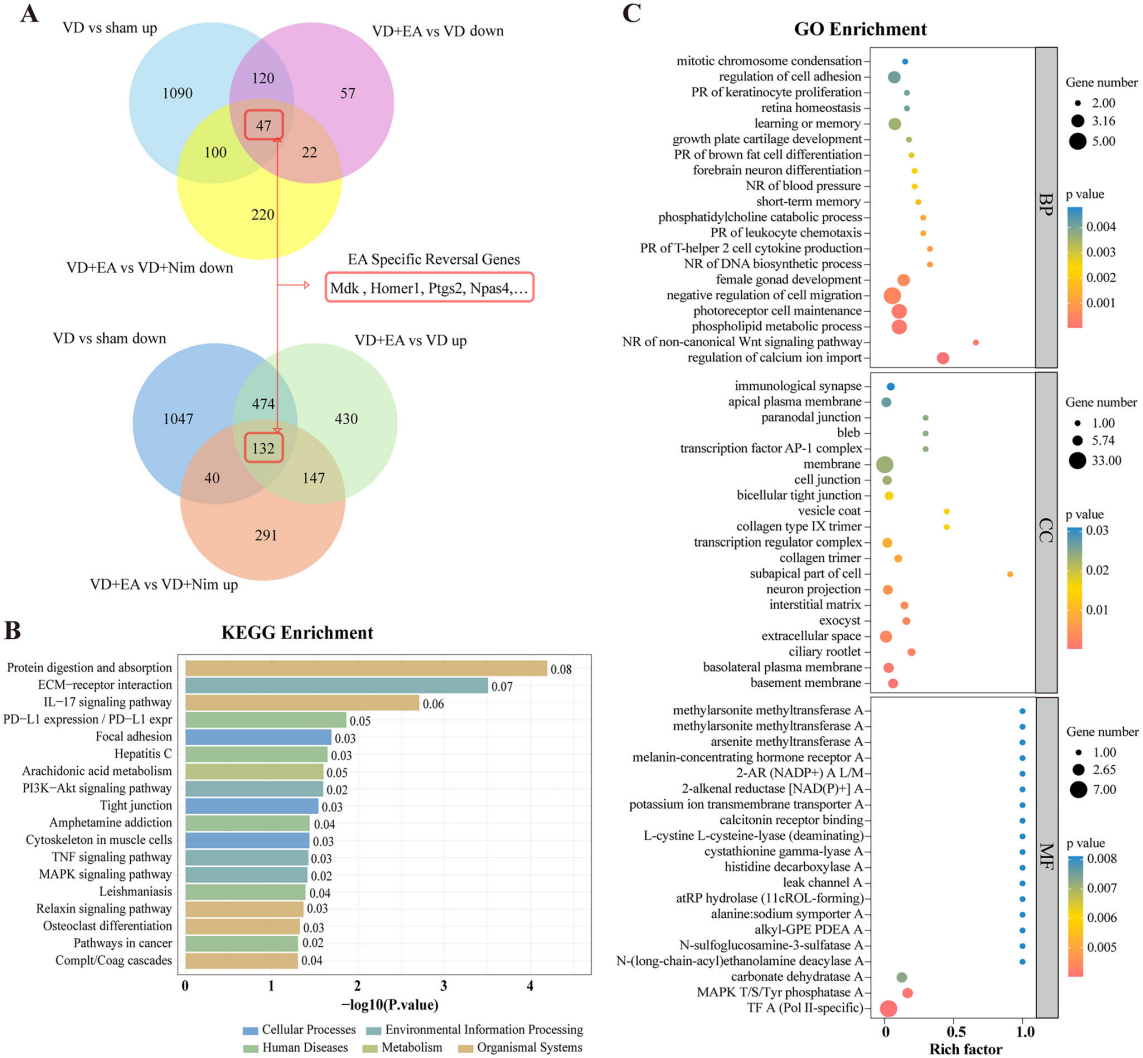
RNA‐seq analysis of transcriptomic alterations in the VD rat hippocampus after EA‐ZSZ treatment. (A) Venn diagrams for selecting target genes for further analysis. The upper panel shows “pathology‐related” genes (upregulated in VD vs. Sham, but downregulated after EA‐ZSZ treatment, with a greater restorative effect than Nimodipine). The lower panel shows “recovery‐related” genes (downregulated in VD vs. Sham, but upregulated after EA‐ZSZ treatment, with a greater restorative effect than Nimodipine). Genes were selected using the criteria *p *< 0.05 and∣log2FC∣ ≥ 1. The union of genes from the two intersections was used for subsequent functional analyses. (B) KEGG pathway enrichment analysis of the union gene set, *p* < 0.05. (C) GO enrichment analysis of the union gene set, *p* < 0.05.

To investigate the signaling pathways underlying the neuroprotective effects of EA‐ZSZ, we performed RNA‐seq on hippocampal tissues (Figure 3A). The heatmap revealed distinct differences between the VD and Sham groups (Figure 3B). Volcano plots illustrated the upregulation of AS‐related inflammatory genes and the downregulation of synaptic repair genes in the VD group compared with the Sham group (Figure 3C). Functional enrichment analyses (KEGG and GO) of the genes altered by VD showed a significant over‐representation of pathways related to inflammatory signaling (including TNF and MAPK signaling), as well as terms associated with the extracellular region/extracellular signaling and growth factor activity (Figure 3D,E). Therefore, we hypothesized that VD is associated with a pro‐inflammatory transition of AS and alterations in synaptic organization within the hippocampus. To identify transcripts potentially involved in the EA‐ZSZ response, we next defined “reversal genes” as VD‐associated DEGs whose expression shifted toward Sham levels following EA‐ZSZ treatment, but not after nimodipine treatment (Figure 4A). Several representative genes followed this reversal pattern, including *Mdk*, *Homer1*, *Ptgs2*, and *Npas4*. Enrichment analyses of this reversal gene set highlighted categories related to synaptic function/plasticity and inflammatory regulation, primarily involving AS secretion (Figure 4B,C), suggesting that EA‐ZSZ is beneficial for ameliorating the synaptic and inflammation‐related gene networks disrupted by VD. Consequently, in subsequent experiments, we assessed the expression of key AS‐associated neurotrophic factors and inflammatory mediators to complement the transcriptomic findings.

### 3.4 EA‐ZSZ Modulates Astrocyte Reactivity and Enhances the Neurotrophic/Inflammatory Microenvironment

**FIGURE 5:**
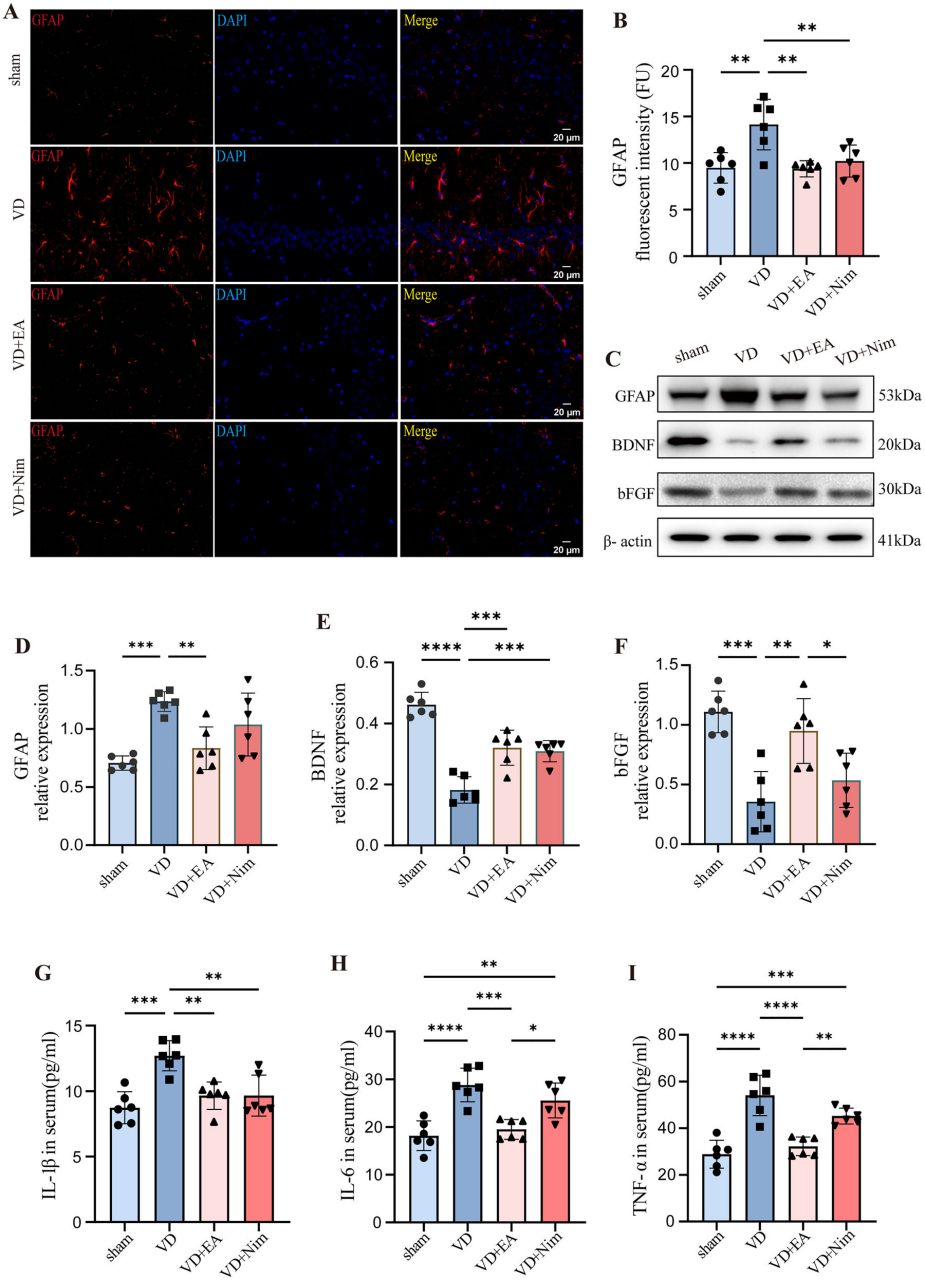
EA‐ZSZ attenuates astrocyte activation and upregulates neurotrophic factors in the rat hippocampal CA1 region and reduces serum inflammatory factor levels. (A) Representative immunofluorescence images of GFAP staining in the hippocampal CA1 region. Scale bar = 20 µm; (B) quantitative analysis of the mean fluorescence intensity of GFAP; (C) representative Western blot bands showing protein expression levels of GFAP, BDNF, and bFGF in the hippocampus; (D–F) quantitative analysis of GFAP, BDNF, and bFGFprotein expression; (G–I) quantitative analysis of IL‐1β, IL‐6, and TNF‐α levels in rat serum. Data are presented as mean ± SD. *p < 0.05, **p < 0.01, ***p < 0.001, compared with each group; *n* = 6 per group.

Immunofluorescence staining and Western blotting analyses showed that the VD‐induced increase in GFAP‐positive ASs and their morphological activation—characterized by enlarged somata and thickened processes—were significantly attenuated in both the VD+EA and VD+Nim groups (Figure 5A, B). Western blot results confirmed that GFAP expression was significantly higher in the VD group compared with the Sham group, but was downregulated following EA‐ZSZ treatment (Figure 5C,D). Furthermore, compared with the Sham group, the VD group showed downregulated expression of neurotrophic factors BDNF and bFGF, while EA‐ZSZ treatment upregulated both factors relative to the VD group, and nimodipine only increased BDNF levels, with no significant effect on bFGF (Figure 5C, E, F). Finally, ELISA assessment of systemic inflammation revealed that serum concentrations of IL‐1β, IL‐6, and TNF‐α were significantly elevated in the VD group compared with the Sham group. Following intervention, the VD+EA group exhibited a significant reduction in the levels of all three cytokines, whereas the VD+Nim group only showed a decrease in IL‐1β levels (Figure 5G–I).

## 4 Discussion

In this study, we demonstrated that EA‐ZSZ significantly alleviates cognitive deficits in a rat model of VD induced by modified 2‐VO. Our findings indicate that EA‐ZSZ exhibits superior efficacy over nimodipine in restoring spatial learning and hippocampal structural integrity. This therapeutic advantage is closely linked to the transcriptomic reprogramming of inflammatory and synaptic networks, specifically through the modulation of the AS–synapse axis.

### 4.1 AS Phenotype and the Inflammatory Microenvironment

ASs are no longer viewed merely as supporting cells but as integral components of the “tripartite synapse,” actively shaping the chemical and physical environment of neuronal synapses (Escartin et al. 2021). Following chronic hypoperfusion, we observed a significant upregulation of GFAP, a hallmark of reactive astrogliosis. This morphological activation coincided with our transcriptomic findings, which showed an enrichment of pathways governing inflammatory responses (e.g., TNF and MAPK signaling). Excessive or maladaptive astrocytic activation often leads to a “pro‐inflammatory” phenotype (A1‐like) that exacerbates synaptic loss and neuronal death (Santos et al. 2025).

Our data revealed that EA‐ZSZ significantly reduced elevated serum levels of TNF‐α, IL‐1β, and IL‐6. Given that TNF‐α is a primary inducer of the neurotoxic A1‐type ASs (Liddelow et al. 2017), this reduction in systemic inflammatory triggers likely facilitates the transition of ASs from a detrimental reactive state to a neuroprotective phenotype. Such a shift is essential for stabilizing the hippocampal microenvironment and limiting chronic neuroinflammation (Li et al. 2022). This is further supported by the attenuated GFAP expression and restored astrocytic morphology observed in the CA1 region, suggesting that EA‐ZSZ reduces the inflammatory milieu identified in our RNA‐seq analysis.

### 4.2 Trophic Support and the Structural Plasticity of Neuronal Synapses

The structural plasticity of neuronal synapses is the biological substrate of cognitive function. Within the hippocampal CA1 region, the maintenance of synaptic connections relies heavily on the availability of neurotrophic factors and a stable secretory microenvironment (Leal et al. 2017). Our transcriptomic analysis identified “reversal genes” such as, *Mdk*, *Homer1*, and *Npas4*. Importantly, the expression levels of these genes showed a strong quantitative consistency with our protein‐level data and functional outcomes. For instance, the upregulation of *Homer1*, a scaffold protein in the PSD, correlates directly with the restored PSD thickness and synaptic curvature observed in our TEM analysis. Similarly, *Npas4*, an activity‐dependent transcription factor, acts as a master regulator of inhibitory‐excitatory balance, providing a genetic blueprint for the improved cognitive performance in the MWM test.

Furthermore, we observed a significant upregulation of BDNF and bFGF following EA‐ZSZ treatment. While both factors are crucial for synaptic repair, they likely play complementary roles: BDNF primarily enhances synaptic transmission and long‐term potentiation by modulating glutamate receptor trafficking, whereas bFGF acts as a potent neuroprotective agent that stabilizes the extracellular matrix and supports the metabolic demands of regenerating synapses. The simultaneous enhancement of these two factors suggests that EA‐ZSZ promotes a “pro‐regenerative” microenvironment through the AS–synapse axis. This enhanced trophic support provides the necessary milieu for the repair of damaged neuronal synapses and the maintenance of synaptic density. This is consistent with prior evidence that acupuncture can upregulate plasticity‐related proteins and protect against ischemic synaptic damage (Xin et al. 2025).

### 4.3 Comparative Superiority and Multi‐Target Synergy of EA‐ZSZ

A noteworthy finding is the mechanistic divergence between EA‐ZSZ and nimodipine. While nimodipine effectively reduced IL‐1β levels, it failed to significantly alter the BDNF/bFGF profile or the GFAP‐positive reactive state of ASs. In contrast, EA‐ZSZ demonstrated a more comprehensive “multi‐target” effect. By simultaneously suppressing systemic neuroinflammation and enhancing the structural and trophic support for neuronal synapses, EA‐ZSZ achieved a more robust restoration of the hippocampal microenvironment. This specificity of “Zhisanzhen” in modulating the AS–synapse axis underscores its advantage in treating complex neurodegenerative pathologies like VD, where single‐target pharmacological interventions often fall short.

### 4.4 Mechanistic Hypothesis of EA‐ZSZ Action

Based on our findings, we propose a potential mechanistic hypothesis for the neuroprotective effects of EA‐ZSZ: EA may act as a systemic regulatory stimulus that initially suppresses the excessive release of pro‐inflammatory cytokines (e.g., TNF‐α and IL‐1β) from both peripheral and central sources. This reduction in the inflammatory “surge” prevents ASs from transitioning into a neurotoxic A1‐like reactive state. Consequently, these stabilized ASs are able to maintain their homeostatic roles, such as the synergistic secretion of neurotrophic factors (BDNF, bFGF, and *Mdk*), which collectively optimize the hippocampal microenvironment to support synaptic structural repair and functional recovery.

### 4.5 Limitations and Future Perspectives

Despite these promising results, several limitations must be acknowledged. First, our transcriptomic analysis utilized an unadjusted *p*‐value threshold (*p *< 0.05) with a fold change ≥ 2 to identify candidate genes. While this approach enabled us to capture a broader spectrum of molecular targets for downstream validation, it increases the risk of false‐positive findings compared to FDR‐adjusted analyses. Second, although our data demonstrate a strong correlation between EA‐ZSZ treatment and the modulation of the AS–synapse axis, establishing a definitive causal link remains to be fully elucidated. We recognize that further evidence—such as the expression analysis of glutamate transporters (e.g., EAAT2/GLT‐1), AS‐specific synaptic proteins (e.g., Thrombospondin‐1), or high‐resolution co‐localization of glial and synaptic markers—is required to fully define the interaction within this axis. Furthermore, the degree to which specific neurotrophic factors directly mediate the behavioral improvements requires validation through loss‐of‐function or rescue experiments (e.g., using chemogenetic tools or cell‐specific knockouts).

Nevertheless, the ability of EA‐ZSZ to normalize coordinated gene networks—rather than isolated factors—aligns with its documented multifaceted properties (Jianyi et al. 2023), positioning it as a potent therapeutic strategy for remodeling the damaged brain microenvironment in VD.

## 5 Conclusion

In summary, our study demonstrates that EA‐ZSZ effectively alleviates cognitive impairment and hippocampal damage in a rat model of VD. Through the integration of transcriptomic profiling and experimental validation, we show that the therapeutic benefits of EA‐ZSZ are closely associated with a comprehensive reprogramming of the AS–synapse axis. By modulating the hippocampal secretory microenvironment—specifically by suppressing pro‐inflammatory cytokines while synergistically upregulating neurotrophic factors like BDNF, bFGF, and Mdk—EA‐ZSZ facilitates the restoration of synaptic structural plasticity and normalizes coordinated molecular networks (e.g., *Homer1* and *Npas4*). These findings support the clinical application of Zhisanzhen and suggest that EA may serve as a promising multi‐target intervention for complex neurodegenerative disorders.

## Author Contributions

**Taotao Zhou**: methodology, investigation. **Sijing Guo**: conceptualization, methodology, data curation, software, writing – original draft, writing – review and editing, formal analysis. **Zhongsheng Tang**: writing – review and editing, funding acquisition, resources. **Qian Liu**: validation, investigation, data curation. **Shihao Lin**: software, validation, visualization. **Qin Hu**: supervision, project administration.

## Funding

This work was supported by the National Natural Science Foundation of China (Grant No. 82360873) and the Science and Technology Plan Project of Guizhou Province (Grant No. ZD(2025)016).

## Ethics Statements

Male SD rats (6–8 wk old and weighing 250 ± 30 g) were purchased from Changsha Tianqin Experimental Animal Breeding Co. Ltd. [Production License No. SCXK (Xiang) 2022‐0011]. All experimental procedures were approved by the Animal Ethics Committee of Guizhou University of Traditional Chinese Medicine (Ethics Approval No. 2024077)and were conducted in accordance with the UK Animals (Scientific Procedures) Act, 1986, EU Directive 2010/63/EU, and the National Research Council's Guide for the Care and Use of Laboratory Animals. All protocols adhered to the Institute of Health standards and complied with the ARRIVE guidelines. Rats were housed in a controlled environment with a 12‐h light/dark cycle, 55% ± 5% humidity, and a temperature of 25 ± 1°C, with an air‐filtration system to maintain optimal conditions.

## Conflicts of Interest

The authors declare no conflicts of interest.

## Data Availability

The raw data supporting the conclusions of this article will be made available by the authors, without undue reservation.
